# The use of semantic similarity measures for optimally integrating heterogeneous Gene Ontology data from large scale annotation pipelines

**DOI:** 10.3389/fgene.2014.00264

**Published:** 2014-08-06

**Authors:** Gaston K. Mazandu, Nicola J. Mulder

**Affiliations:** Computational Biology Group, Department of Clinical Laboratory Sciences, Institute of Infectious Disease and Molecular Medicine, University of Cape TownCape Town, South Africa

**Keywords:** functional annotation, Gene Ontology annotation, annotation pipeline, manual annotation, electronic annotation

## Abstract

With the advancement of new high throughput sequencing technologies, there has been an increase in the number of genome sequencing projects worldwide, which has yielded complete genome sequences of human, animals and plants. Subsequently, several labs have focused on genome annotation, consisting of assigning functions to gene products, mostly using Gene Ontology (GO) terms. As a consequence, there is an increased heterogeneity in annotations across genomes due to different approaches used by different pipelines to infer these annotations and also due to the nature of the GO structure itself. This makes a curator's task difficult, even if they adhere to the established guidelines for assessing these protein annotations. Here we develop a genome-scale approach for integrating GO annotations from different pipelines using semantic similarity measures. We used this approach to identify inconsistencies and similarities in functional annotations between orthologs of human and *Drosophila melanogaster*, to assess the quality of GO annotations derived from InterPro2GO mappings compared to manually annotated GO annotations for the *Drosophila melanogaster* proteome from a FlyBase dataset and human, and to filter GO annotation data for these proteomes. Results obtained indicate that an efficient integration of GO annotations eliminates redundancy up to 27.08 and 22.32% in the *Drosophila melanogaster* and human GO annotation datasets, respectively. Furthermore, we identified lack of and missing annotations for some orthologs, and annotation mismatches between InterPro2GO and manual pipelines in these two proteomes, thus requiring further curation. This simplifies and facilitates tasks of curators in assessing protein annotations, reduces redundancy and eliminates inconsistencies in large annotation datasets for ease of comparative functional genomics.

## 1. Introduction

The development of fast and relatively inexpensive sequencing technologies has yielded complete genome sequences of thousands of organisms. Several sequence databases store these sequences, including GenBank Benson et al. (2009), Ensembl Flicek et al. (2010); Fernández-Suárez and Schuster (2010); Spudich and Fernández-Suárez (2010), NCBI (Pruitt et al., 2005; Sayers et al., 2009) and the UniProt database, which is an integrated repository of protein sequence and function created by joining the information contained in the Swiss-Prot, TrEMBL, and PIR proteins databases (Jain et al., 2009; UniProt-Consortium, 2010). In these databases, an increased deficiency in functional annotation was observed for many sequenced proteins as approximately 20–50% of proteins within a genome were still labeled “unknown,” “uncharacterized” or “hypothetical” (Mazandu and Mulder, 2012). Thus, several annotation pipelines, including experimental and electronic, were developed to functionally characterize these proteins. Subsequently, the Gene Ontology (GO) (GO-Consortium, 2012) arose to organize and unify biology and information about genes and proteins shared by different organisms, and emerged as one of the dominant and most popular functional classification schemes for functional annotation of genes and their products.

Many annotation pipelines were developed to predict or assign functions to proteins using GO terms from the three different ontologies of GO, namely Biological Process (BP), Molecular Function (MF), and Cellular Component (CC). These include electronic annotation methods, such as Ensembl Compara, InterPro, UniProtKB/Swiss-Prot Keywords (SPKW), UniProtKB-Subcellular Location (SPSL), UniPathway, Enzyme Commission (EC), and High-quality Automated and Manual Annotation of Microbial Proteomes (HAMAP), and manual annotation efforts, such as the Gene Ontology Annotation project, the Reference Genome Annotation Initiative (The Reference Genome Group of the Gene Ontology Consortium, 2009), and Cardiovascular (www.ebi.ac.uk/GOA/CVI) and Renal (www.ebi.ac.uk/GOA/kidney) Gene Ontology Annotation Initiatives. The Gene Ontology Annotation (GOA) project at the European Bioinformatics Institute (EBI) commits to integrating these protein annotations into a single set of high-quality electronic and manual associations (annotations) of GO terms to UniProt Knowledgebase (UniProtKB) entries. Most of the data is generated from the conversion maps, namely SPKW2GO, SPSL2GO, EC2GO, HAMAP2GO, UniPathway2GO and InterPro2GO, which themselves are manually curated to ensure high-accuracy annotations from the electronically inferred GO annotation set.

Producing high-quality and accurate GO annotations is challenging, as manual annotation is a slow and expensive process and the number of manual annotations available for a particular genome is usually far fewer than those produced by electronic annotation pipelines. To improve the annotation quality, the GOA project does manual GO curation following standards set by the GO Consortium (http://www.geneontology.org/GO.annotation.conventions.shtml). However, different annotation pipelines can differ widely in their specific procedures (annotation algorithms, confidence thresholds, etc.) and this increases the heterogeneity across a gene set, thus making curators' tasks difficult. Furthermore, considering the structure of GO, the set of associations produced by GOA is often redundant and subjected to potential mismatches and inconsistencies (Dolan et al., 2005). Fortunately, the hierarchical structure of the GO enables the assessment of the GO term closeness using semantic relationships between terms. These semantic relationships between terms have been used to set up semantic similarity tools that enable efficient exploitation of the enormous corpus of biological knowledge embedded in the GO directed acyclic graph (DAG) structure by comparing GO terms and proteins at the functional level (Mazandu and Mulder, 2013a).

The use of semantic relationships between GO terms enables the quantification of GO term specificity through measurements of information content (IC) values in the GO DAG (Mazandu and Mulder, 2013b). These GO term IC values are used to evaluate semantic similarity scores between GO terms and annotated proteins, reflecting the closeness between two concepts in the GO DAG. Several semantic similarity measures have been introduced (Mazandu and Mulder, 2012, 2013a,b) and used in different biological applications, such as gene clustering, gene expression data analysis, prediction and validation of molecular interactions, and disease gene prioritization (Mazandu and Mulder, 2013a). In the context of current high-throughput biological technologies, these measures may be used to set up novel bioinformatics approaches that enable the efficient use of the GO DAG structure for integrating annotations from multiple sources. Here we use these semantic similarity measures as tools to develop an efficient, large-scale approach that enables “optimal” integration of protein GO annotations from heterogeneous annotation pipelines or different sources, thus reducing redundancy and eliminating inconsistencies in the integrated annotation datasets for ease of comparative functional genomics.

## 2. Materials and methods

In order to compare different annotation pipelines, to filter an annotation dataset or to integrate annotations from different pipelines, we define GO annotation quality assessment measures based on GO semantic similarity scores. These measures are applied to *Drosophila melanogaster* (fruitfly) and human GO annotations as a demonstration of the variety of applications. We assess GO annotations assigned by InterPro, which are electronically inferred with IEA (Inferred from Electronic Annotation) as the evidence code, in comparison with the manual and experimental annotation in FlyBase (Tweedie et al., 2008). In addition, we compare annotations in Flybase to orthologous proteins from human to assess the quality of GO annotation between different annotation groups and we integrate electronically inferred and experimental annotations for *Drosophila melanogaster* proteins.

### 2.1. GO annotation quality assessment measures

Currently electronic annotation pipelines dominate manual annotation in terms of number of annotations available for a particular genome and this is the most likely future trend. The GO annotations produced by these electronic annotation pipelines represent more than 98% of the GOA dataset, but the mapping filters used are, in most cases, manually curated, which increases confidence levels of these annotations, even though they still receive the IEA evidence code. In this context, the GO annotation quality for a proteome or a set of proteins should mainly be measured by the specificity or the level of detail of GO annotations used, the consistency of these annotations in terms of similarity between terms used and the non-redundancy of the annotation set. We used semantic similarity measures between terms in the GO DAG to derive these different GO annotation quality assessment measures, as described below.

#### 2.1.1. Fuzzy non-redundant set of annotations for a gene

Given a gene *g* with *T^X^_g_* its set of GO terms in the ontology *X*, a term *t∈ T^X^_g_* is redundant in *T^X^_g_* when it contributes to the specification of its descendant term *s∈ T^X^_g_* based on the semantic similarity between them, in which case the score is greater than a given threshold δ ≥ 0. This threshold score provides the semantic similarity degree at which an ancestor term is considered to semantically reflect in the specification of its descendant or a child term consistently includes the ancestor term under consideration in its specification. The set 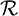
^*X*^_*g*_ of redundant annotations for the gene *g* is given by 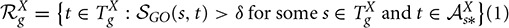 where 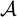
^X^_s*_ denotes the set of ancestors of the term *s* in the ontology *X* and 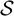
*_GO_(s,t)* is the semantic similarity score between GO terms *s* and *t* retrieved from the following formula (Mazandu and Mulder, 2013b): 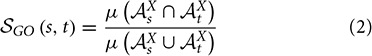 where 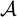
*^X^_x_* = 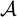
^X^_x*_∪{x}, μ(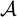
*^X^_s_* ∩ 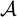
*^X^_t_*)≥ 0 and μ(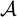
*^X^_s_* ∪ 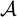
*^X^_t_*) > 0 are measures of the commonality between and of the description of 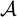
*^X^_s_* and 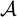
*^X^_t_*, respectively.

The set 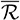
*^X^_g_* of non-redundant terms annotating the gene *g* is the complementary set of 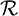
*^X^_g_* in *T^X^_g_*, i.e.,

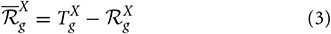



If δ = 0, the set 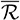
*^X^_g_* is referred to as the set of strict non-redundant terms annotating the gene *g*, which represents the set of terms annotating a gene at a high level. The functional redundancy score 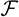
*_R_(g)* of the gene *g* is given by 
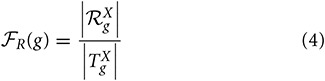
 where |*T*| is the number of terms in the set *T*. 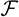
*_R_(g)* represents the proportion of terms in the set of terms annotating the gene *g* that are ancestors implicitly included to other term specification in that set based on the semantic similarity between them. Thus, the term “fuzzy” is used to map the fact that the consideration of an ancestor term in the specification of its descendant is no longer a two-valued logic yes (1) or no (0) but now depends on the semantic similarity score between them.

#### 2.1.2. Assessing functional specificity of a gene

A given gene can perform several functions or be involved in several processes. In this case, the gene is annotated by a set of GO terms. The specificity of each term assessing its informativeness depends on its position in the GO structure and the deeper the term is in the DAG structure the more specific or informative the term is. This indicates that the closer or the more similar to the leaf term (term without a child term) the more specific or informative the term is. The specificity score of an annotated gene depends on the specificity of terms used to annotate the gene and is the average of specificity scores of terms in the set of strict non-redundant terms annotating the gene. Thus, for a given gene *g*, its functional specificity score 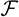
*_S_(g)* is assessed by measuring how similar its GO annotations are to leaf terms of the GO DAG connected to the set 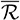
*^X^_g_* of strict non-redundant terms annotating *g*. This functional specificity score is computed as follows: 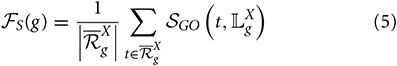 where 𝕃*^X^_g_* is the set of all leaves of the GO DAG connected to all terms in 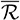
*^X^_g_*. The higher this score is the more informative the set of terms annotating the gene under consideration is.

#### 2.1.3. Quantifying gene annotation consistency

In general, different annotations of a given protein are expected to be less diverse or more coherent (Defoin-Piatel et al., 2011) with minimum variability among them. In order to quantify the coherence among different annotations of a gene or protein, we compute the annotation consistency score 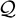
*_C_(g)* for a gene *g* as follows: 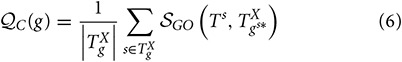 where *T^s^* = *{s}* and T^X^_g^s*^_ = T^X^_g_ −{s} with *T^X^_g_* the set of GO terms in the ontology *X* annotating *g* and 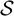
_GO_(T^s^, T^X^_g^s*^_) is the semantic similarity between two sets of GO annotations, singleton *T^s^* and T^X^_g^s*^_, and can be computed using any semantic similarity model (Mazandu and Mulder, 2013a). The gene annotation consistency score ranges between 0 and 1, and enables the detection of annotation inconsistencies. This score is set to 1 for a gene with one annotation and the lowest value of annotation consistency score is an indication of possible annotation error for the gene under consideration or alternatively it indicates that the gene is multi-functional at the molecular level or involved in several biological processes. Such gene annotations should be subjected to further investigation to check their accuracy.

### 2.2. Scoring gene annotation matches and mismatches between pipelines

Different annotation pipelines are likely to lead to different GO annotations for a given gene. This is due to several reasons (Dolan et al., 2005), including the GO structure itself, incomplete annotation and annotation errors. Difference in annotation for a gene or protein related to the GO structure or incomplete annotation is often a consequence of insufficient knowledge about either the protein being annotated or the term being used to annotate the protein under consideration. These differences and even those due to annotation error from manual annotation assignment can be resolved and corrected by the curator. However, if the annotation assignment has been inferred electronically, the annotation error may be hard to correct since the source of the error may not be local or the cause of the error is far from the point where it is being detected as a result of the propagation of annotation errors from protein databases (Devos and Valencia, 2000).

Currently, the level of gene or protein annotation matches or mismatches can be quantitatively scored using semantic similarity measures (Mazandu and Mulder, 2013a) by computing a semantic similarity score 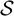
*_GO_*(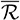
*^X^_p^1^_*, 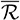
*^X^_p^2^_*) between strict non-redundant sets of GO annotations 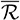
*^X^_p^1^_* and 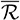
*^X^_p^2^_* provided by different annotation pipelines for a protein *p*. Thus, the score of an annotation match ω(*p*) for a protein *p* is given by: 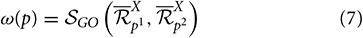 and the score of an annotation mismatch ω(*p*) is computed as follows:

(8) $$\bar{\omega }(p)=1-\omega (p)$$

for a given protein *p*, two annotation pipelines are completely in agreement if ω(*p*) = 1 or ω(*p*) = 0 and completely in disagreement if ω(*p*) = 1 or ω(*p*) = 0. The complete disagreement between two annotation pipelines about annotations assigned to a protein is an indication of an annotation error from one pipeline or a protein is multi-functional and the two pipelines under consideration are unable to identify multi-annotations or diversified annotations for multi-functional proteins, in which case further curation is required.

### 2.3. Comparing different annotation pipelines

In this study, we use functional specificity, redundancy and consistency scores to quantitatively assess GO annotation quality of an annotation pipeline or dataset. The “optimal” pipeline or dataset is that producing good annotation quality scores, enabling comparison of different pipelines or datasets. In addition, when comparing different datasets, matches, mismatches and missing annotations are checked or identified. Here, we applied these different scores to human and fruitfly proteomes annotated by InterPro2GO and manual pipelines using the GO-universal metric (Mazandu and Mulder, 2012) as a semantic similarity model. However, this approach is applicable to any dataset and researchers can adapt it to any semantic similarity model. This is useful for curators and end-users of datasets to detect issues related to different datasets as quantitative quality assessment is crucial to the comparative functional genomics community.

### 2.4. Integrating GO annotations and filtering gene annotation datasets

Integrating GO annotations from different pipelines or filtering gene annotation datasets consists essentially of reducing redundancy in the integrated dataset produced. There are two types of redundancy detected in a given genome or annotation data set. The first type, referred to as type I redundancy, is due to the “true path” rule in the hierarchical structure of the GO DAG according to which a child term contains all features of its ancestor terms. The second one, referred to as type II redundancy, is due to the GO evidence code or the source (reference) of the annotation. The type II redundancy occurs when the same annotation is generated by different pipelines (electronic or manual/experimental) for a given protein and this is mainly reflected in the size of the file storing the dataset produced. We illustrate these different types of redundancy on the protein *E3 ubiquitin-protein ligase Topors* (Q9NS56) with GO MF annotations retrieved from the GOA database and shown in Table 1. In this GO annotation set, the GO term *DNA topoisomerase binding* (GO:0044547) is a descendant of the term *protein binding* (GO:0005515), and thus contains all biological specifications of this ancestor and including this ancestor (GO:0005515) in the set produces a type I redundancy. The GO term *ubiquitin-protein transferase activity* (GO:0004842) or *SUMO ligase activity* (GO:0019789), for example, was assigned to this protein by different sources or by the same source using different methods and recorded in the annotation file. This yields a type II redundancy, which only reflects in the file storing these annotations but not in any analysis using these annotations.

**Table 1:** **GO annotations of the protein *E3 ubiquitin-protein ligase Topors* (Q9NS56)**.

**GO ID**	**GO term**	**Level**	**Evidence code**	**Source**
GO:0008270	Zinc ion binding	6	IEA	InterPro
GO:0004842	Ubiquitin-protein transferase activity	6	IMP	UniProt
GO:0004842	Ubiquitin-protein transferase activity	6	IDA	BHF-UCL
GO:0004842	Ubiquitin-protein transferase activity	6	IDA	UniProt
GO:0019789	SUMO ligase activity	6	IDA	UniProt
GO:0019789	SUMO ligase activity	6	IMP	UniProt
GO:0044547	DNA topoisomerase binding	4	IPI	UniProt
GO:0003677	DNA binding	4	IDA	UniProt
GO:0005515	Protein binding	2	IPI	UniProt
GO:0003823	Antigen binding	2	IPI	UniProt

*These annotations were retrieved from GOA-human gene association from the GOA database and level represents the maximum number of links from the root to the GO term in the GO DAG, assuming that the root of each ontology is located at level 0. Evidence codes IEA, IMP, IPI, and IDA stand for Inferred from Electronic Annotation, Inferred from Mutant Phenotype, Inferred from Physical Interaction and Inferred from Direct Assay, respectively*.

Different sources may also annotate a protein with different GO terms but which are very similar in the context of the GO DAG, resulting in type I redundancy. One can annotate the protein to more specific terms in the same path or to sibling terms, i.e., sharing a direct parent. Lack of more complete biological knowledge about the protein under consideration results in the pipeline annotating it with a more general term. This redundancy issue can be solved through semantic similarity scores between terms in the GO-DAG, which are related when using a given ancestor term for its descendant specification (Mazandu and Mulder, 2012). Thus, the ancestor term is retained only when it is unable to directly contribute to its descendant term specification based on the semantic similarity between them, in which case the score is lower than the agreement level or threshold (Mazandu and Mulder, 2013a).

## 3. Results

In order to compare different annotation pipelines, to filter an annotation dataset or to integrate annotations from different pipelines, we define GO annotation quality assessment measures based on GO semantic similarity scores (see Materials and Methods Section). These quality assessment measures include the specificity or the level of detail of GO annotations used, the consistency of these annotations in terms of similarity between terms used, the non-redundancy of the annotation set and annotation match and mismatch scores. These measures are defined using an abstract semantic similarity measure, but here we are using the GO-universal metric (Mazandu and Mulder, 2012) when applied to the *Drosophila melanogaster* (fruitfly) and human proteomes with GO annotations manually assigned with the following GO evidence codes (Experimental category): Inferred from Experiment (EXP), Inferred from Direct Assay (IDA), Inferred from Physical Interaction (IPI), Inferred from Mutant Phenotype (IMP), Inferred from Genetic Interaction (IGI) and Inferred from Expression Pattern (IEP), and annotations originating from InterPro2GO, referred to as electronic inference.

For *Drosophila melanogaster*, available data from the UniProt database shows 60887 total entries (proteins coding genes), but only totals of 11046, 12048, and 10030 proteins are annotated with respect to the BP, MF, and CC ontologies, respectively, as extracted from the latest version of GOA UniProt (version 130), released on 15 April, 2014 (http://www.ebi.ac.uk/GOA/proteomes). From these totals, only 7299, 3495, and 4860 entries contain annotations manually assigned with respect the BF, MF, and CC ontologies, respectively. Among annotations inferred electronically, a total of 3195, 6055, and 2276 proteins have annotations inferred electronically using InterPro2GO mappings for BP, MF, and CC ontologies, respectively. Similarly, for 47592 total reviewed entries of human proteome from UniProt, 29844, 36177, and 31683 proteins are characterized with respect to the BP, MF, and CC ontologies, respectively, among which 6507, 8665, and 7416 entries contain annotations manually assigned, and 12422, 21989, and 8725 entries with annotations inferred electronically using InterPro2GO mappings.

### 3.1. Comparing manual and interpro annotations

**Figure 1:**
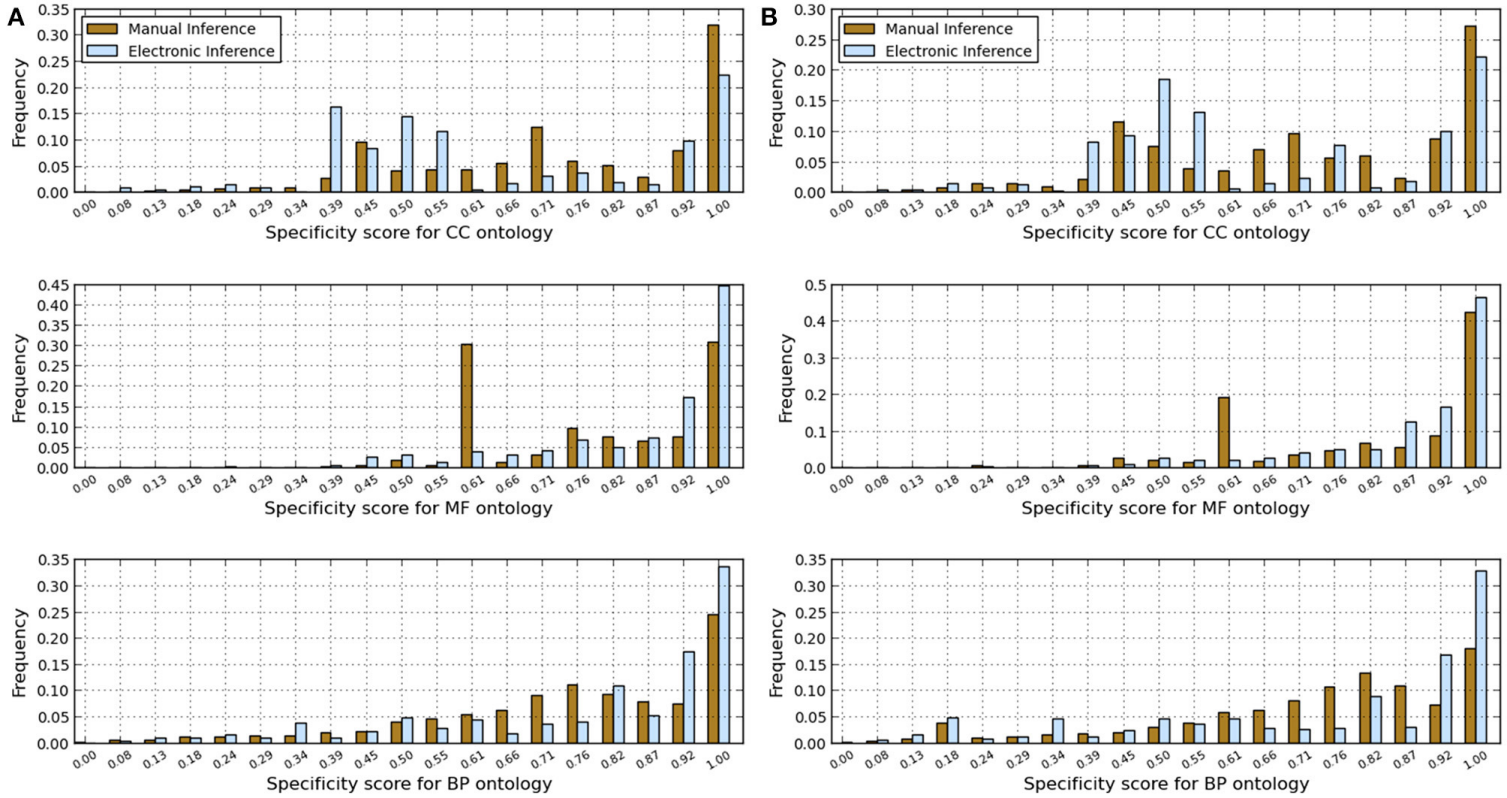
**Comparison of annotations inferred manually and electronically in human and fruitfly genomes in terms of annotation specificity score computed using the GO-universal metric**. **(A)** Human genome. **(B)** Fruitfly genome.

**Figure 2:**
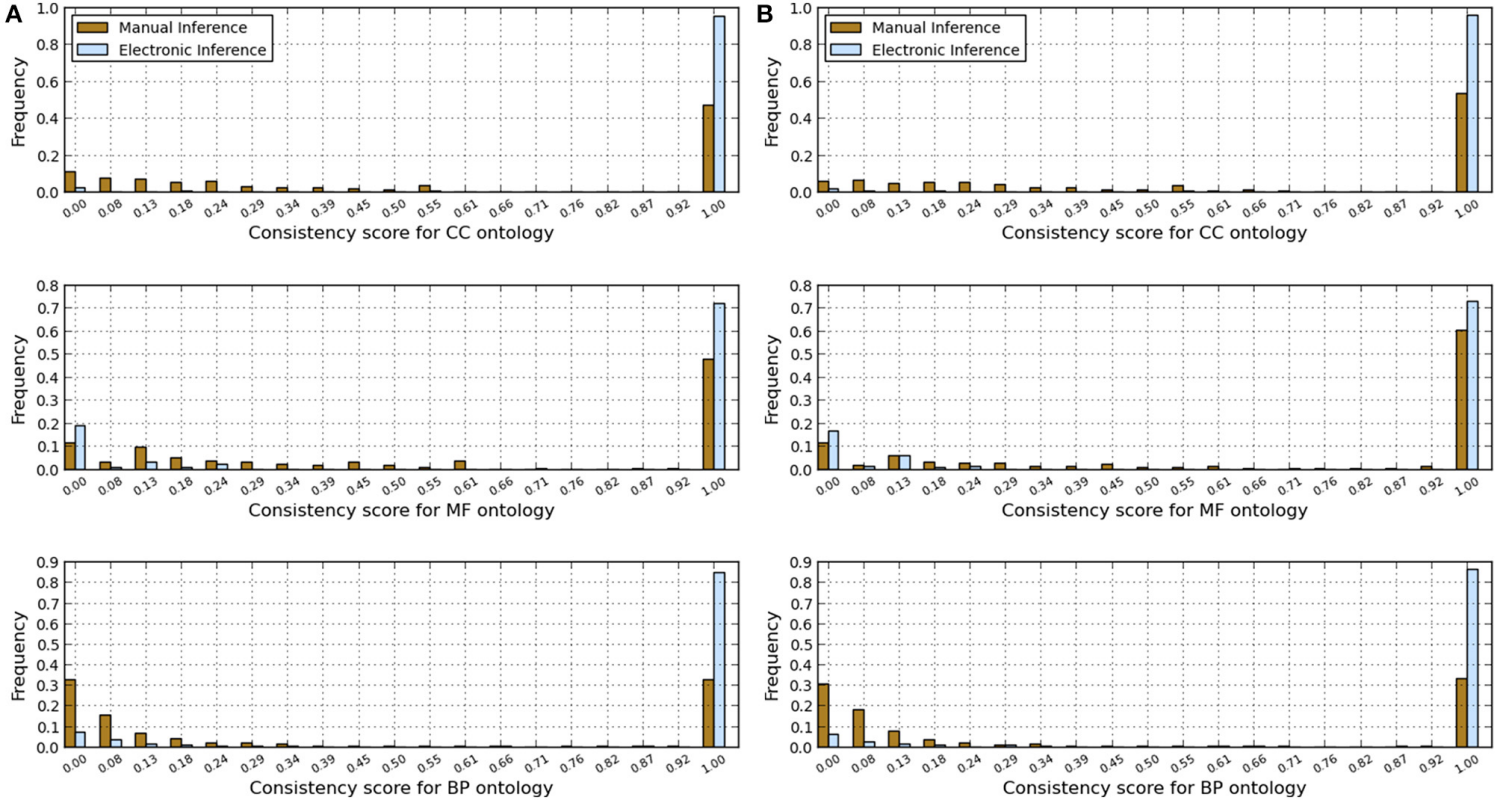
**Comparison of annotations inferred manually and electronically in human and fruitfly genomes in terms of annotation consistency score computed using the GO-universal metric**. **(A)** Human genome. **(B)** Fruitfly genome.

**Figure 3:**
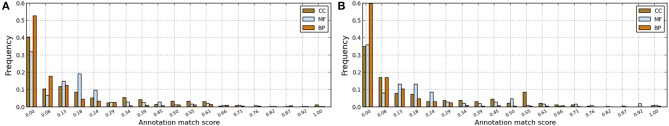
**Annotation matches between manual and electronic pipelines for human and fruitfly genomes with scores computed using the GO-universal metric**. **(A)** Human genome. **(B)** Fruitfly genome.

We analyzed the redundancy in the manual and InterPro2GO pipelines using different confidence levels (0.0, 0.3, and 0.7), the specificity, consistency of terms obtained from InterPro and manual pipelines, and annotation matches (mismatches) between these two pipelines using the GO-universal metric (Mazandu and Mulder, 2012) to compute similarity between terms in a given ontology. Results are shown in Table 2 for redundancy, Figures 1, 2 for annotation specificity and consistency, respectively, and Figure 3 for annotation matches in each organism under consideration.

**Table 2:** **Percentage redundancy of manual and electronic pipelines for different confidence levels**.

**Confidence**	**Genome**	**Biological process**			**Molecular function**			**Cellular component**		
		**EXP**	**IPR**	**AEC**	**EXP**	**IPR**	**AEC**	**EXP**	**IPR**	**AEC**
0.0	Human	22.70	0.00	22.32	28.01	0.00	12.66	22.09	0.00	18.11
	fruitfly	29.02	0.00	27.08	18.31	0.00	13.45	20.72	0.00	17.18
0.3	Human	16.74	0.00	17.44	26.04	0.00	11.63	10.06	0.00	8.89
	fruitfly	20.30	0.00	19.65	16.31	0.00	12.16	10.17	0.00	8.52
0.7	Human	11.20	0.00	11.22	6.08	0.00	4.22	2.28	0.00	1.91
	fruitfly	11.02	0.00	11.40	5.04	0.00	6.74	3.89	0.00	3.41

*The confidence level of 0.0 refers to the strict non-redundancy which consists of using the “true path” rule of the GO structure to identify the ancestor of a term as redundant annotation, but for confidence level of 0.3 and 0.7, a term ancestor is considered to be a redundant annotation for a protein if their semantic similarity score is 0.3 and 0.7, respectively. EXP, IPR, and AEC stand for manual, electronic pipelines and considering all evidence codes, respectively*.

These results indicate that electronic inference (InterPro2GO) produces non-redundant and more consistent annotations than the manual pipeline for different ontologies (Table 2 and Figure 2), and this electronic pipeline also produces more specific annotations for BP and MF ontologies, but not for the CC ontology for which the manual pipeline provides more specific annotations compared to the electronic inference (Figure 1). However, it has been previously reported (Mazandu and Mulder, 2012) that electronic mapping annotations tend to be to higher level GO terms compared to manual pipelines. Furthermore, the lower annotation consistency displayed by the manual pipeline (Figure 2) may be due to the fact that this pipeline is better equipped to identify multi-annotations or diversified annotations for multi-functional proteins. This shows that the only effective way to assign annotations to uncharacterized proteins is the combination of manual and electronic inference (Mazandu and Mulder, 2011). These different results reveal that an optimal integration and filtration of annotations obtained from different pipelines may enable high-quality annotations for a genome annotation. For the two proteomes under consideration, the results in Table 2 show that using the GO-univesal metric for filtering these proteome annotations reduces redundancy in human annotation data up to 22.32, 12.66, and 18.11% and in fruitfly annotation data up to 27.08, 13.45, and 17.18% with respect to the BP, MF and CC ontologies, respectively.

In order to assess annotation coherence between electronic and manual pipelines, we compute functional similarity scores between annotations of a given protein annotated by these two pipelines. Thus, we used the formula (6) and the Best Match Average (BMA) measure (Mazandu and Mulder, 2012, 2013a,b) to score functional similarity between the two sets of annotations for a given protein and results are displayed in Figure 3. These results generally show low annotation matches for several proteins with annotations from the two pipelines (electronic and manual) in the two genomes under consideration for BP, MF, and CC ontologies. Possible reasons include protein mis-annotations, the inability of a given pipeline to identify an annotation for a given protein (missing annotations) and the use of more general GO terms for the manual pipeline and more specific terms for the electronic pipeline (see Figure 1). Once again, these results indicate that the integration of annotations from multiple pipelines can provide more accurate annotations and quality control for functional genomics data.

### 3.2. Assessing fruitfly-human ortholog functional similarity

We compared annotations of ortholog proteins between fruitfly and human to check for annotation equivalence as ortholog proteins share common evolutionary processes and are thought to maintain similar functions (Mazandu and Mulder, 2012). Based on this principle, known as “ortholog” conjecture (Nehrt et al., 2011), electronic annotation pipelines, such as Ensembl Compara, arose in order to transfer annotations between ortholog proteins between different species. In this study, we check for matches and missing annotation for every ortholog protein pair. Ortholog protein pairs were retrieved from the Ensembl website (Flicek et al., 2010; Kinsella et al., 2011) at http://www.ensembl.org using biomart, and GO-association data were downloaded from the GOA site (Dimmer et al., 2012).

**Figure 4:**
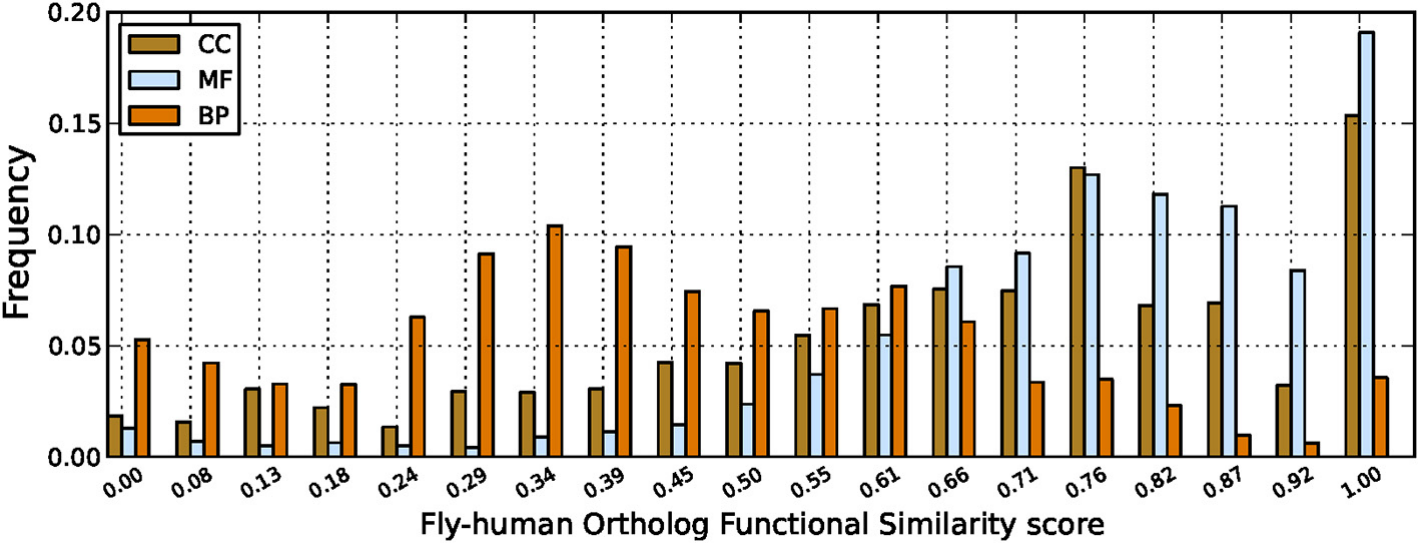
**Fruitfly-human ortholog functional similarity scores**. Comparing GO annotations for ortholog protein pairs between human and fruitfly genomes with scores computed using the GO-universal metric.

From the list of ortholog protein pairs, we have considered those pairs with high confidence according to Ensembl in lists of reviewed proteins (Swiss-Prot) for these two genomes from the UniProt database. We ended up with a list of 3346 ortholog protein pairs with 1988 and 2949 proteins in fruitfly and human proteomes (Ensembl uses a one to many ortholog relationship), respectively, and different results obtained are shown in Table 3 and Figure 4 for different ortholog annotation features and functional similarity scores. The main result in Table 3 is the missing annotations in both genomes for 371, 319, and 485 ortholog proteins in BP, MF and CC ontologies, respectively. This lack of annotations for ortholog proteins in one genome and characterized in another genome is partly to incomplete knowledge, and elicits the need for further curation of existing information about these specific organisms.

**Table 3:** **General features of fruitfly-human ortholog proteins in terms total number of ortholog proteins with GO annotations in BP, MF, and CC ontologies for different genomes under consideration**.

	**Biological process**			**Molecular function**			**Cellular component**		
	**Fruitfly**		**Human**	**Fruitfly**		**Human**	**Fruitfly**		**Human**
Annotated ortholog	1766		2674	1678		2669	1682		2752
Annotated ortholog pair		2866			2829			2759	
Uncharacterized ortholog pair		109			198			102	
Missing annotation ortholog	180		191	95		224	103		382

Results in Figure 4 show high functional similarity between protein orthologs as expected, especially for the MF and CC ontologies. Orthologs with very low functional similarity based on their GO annotations have also been detected, especially for the BP ontology, and this is not in agreement with the belief about function conservation between orthologs. There are several possible reasons for this, including protein mis-annotations, the use of more general GO terms for one and more specific terms for the other protein, or simply the lack of relevant biological knowledge about these proteins, thus requiring further curation of existing knowledge in these systems as pointed out previously. In particular, for the BP ontology describing broad biological goals for genes and proteins, this could be an indication of missing annotations in both orthologs as the differing terms may not be conflicting processes so it may be that the other terms are correct, but have just not yet been added, or they may be organism specific (Mazandu and Mulder, 2012).

### 3.3. Discussion

Worldwide DNA sequencing efforts have led to a rapid increase in sequence data in the public domain, but also the lack of functional annotations for many newly sequenced genes and their predicted proteins. Thus, several genome annotation pipelines were set up to assign or predict functions for the sequenced genes. Even though these annotation pipelines widely adopted the Gene Ontology (GO) as an efficient and standardized functional scheme, the specific procedures followed by each annotation pipeline varies. This leads to an increased heterogeneity created by different granularity of GO annotations across a genome or gene set, rendering curators' tasks tedious and challenging. In this study, we have introduced a large-scale approach that allows the integration of protein GO annotations from different pipelines using GO semantic similarity measures for efficient exploitation of biological knowledge embedded in the GO structure.

We have defined a set of annotation quality assessment measures using an abstract semantic similarity measure and producing a very general approach that can use any semantic similarity measure for filtering and integrating functional annotations from multiple pipelines. We have applied this approach to the manual and InterPro2GO annotation pipelines for the *Drosophila melanogaster* (fruitfly) and human proteomes for different ontologies. In the context of the Gene Ontology, several semantic similarity measures have been introduced and used in different biological applications. These measures are being used to set up novel bioinformatics approaches that enable the efficient use of the GO DAG structure in protein functional analysis, function prediction and assignment. Here, we used the GO universal metric as a semantic similarity measure and results showed that this integrative model may enable high-quality and accurate annotations for genome annotation, reducing redundancy and eliminating inconsistencies in the integrated annotation datasets for ease of comparative functional genomics.

We have assessed GO annotations manually assigned (manual pipeline) with the following GO evidence codes (Experimental category): Inferred from Experiment (EXP), Inferred from Direct Assay (IDA), Inferred from Physical Interaction (IPI), Inferred from Mutant Phenotype (IMP), Inferred from Genetic Interaction (IGI) and Inferred from Expression Pattern (IEP), and compared them to those originating from InterPro (InterPro2GO pipeline). Results revealed that, in general, the InterPro2GO (electronic) pipeline produces non-redundant and more consistent annotations than the manual pipeline due to the fact that the manual pipeline is possibly assigning diversified annotations to multi-functional proteins. This suggests that an efficient integration and filtration of annotations from different pipelines would enable high-quality non-redundant annotation in genome annotation. In the case of human and fruifly annotation datasets, an efficient integration of annotation pipelines can reduce redundancy up to 27 and 22% (see Table 2), respectively, and identify mismatches between some protein annotations from these pipelines (see Figure 3) and incoherence or missing annotations for some orthologs in the two proteomes under consideration (see Table 3 and Figure 4), requiring further curation of existing knowledge. Such an approach is useful for the comparative genomics community, simplifies tasks of curators and should advance comparative functional genomics research.

## Author contributions

Nicola J. Mulder conceived and supervised the project, and finalized the paper. Gaston K. Mazandu analyzed, designed, and implemented the method and wrote the paper. Gaston K. Mazandu and Nicola J. Mulder analyzed data and read and approved the final paper and Nicola J. Mulder approved the production of this paper.

## Funding

This work has been supported by the National Research Foundation (NRF) in South Africa, University of Cape Town.

### Conflict of interest statement

The authors declare that the research was conducted in the absence of any commercial or financial relationships that could be construed as a potential conflict of interest.
